# The Treatment of Cosmetic Lower Eyelid Adverse Events After Injection of Hyaluronic Acid Gel Fillers

**DOI:** 10.1093/asjof/ojae026

**Published:** 2024-04-23

**Authors:** Steven Fagien

## Abstract

**Background:**

Nonsurgical blepharoplasty has been considered as an unmet need particularly for the large potential population of patients who are not interested in or ready for surgery and is generally performed by all providers who offer injectable agents in their practice. Although favorable outcomes can be achieved in most areas of the face, the lower eyelid has shown to be less forgiving and a source of overwhelming unsatisfactory outcomes. Although providers often disclose to their patients that undesirable cosmetic adverse events can be reversed with the injection of hyaluronidase, a larger experience has suggested that this application is not always successful.

**Objectives:**

The author describes an algorithm for the resolution of refractory adverse events using a combination of intraoperative, high-dose, hyaluronidase with lower blepharoplasty.

**Methods:**

From June 2016 to present, 70 consecutive patients, 65 females and 5 males, with ages ranging from 31 to 76 years, presented with unsatisfactory results after they received an injection of a hyaluronic acid (HA) product elsewhere. They were treated with lower blepharoplasty in conjunction with intraoperative “high-dose” transconjunctival hyaluronidase injections.

**Results:**

All patients showed significant but varied improvement after this treatment when, in most situations, in-office, percutaneous injection of hyaluronidase was insufficient.

**Conclusions:**

The combination of intraoperative injection of high-dose hyaluronidase and (upper and/or) lower blepharoplasty can lead to satisfactory outcomes in patients with chronic lower eyelid edema after HA gel injections to the lower eyelid prove ineffective.

**Level of Evidence: 4:**

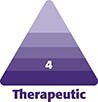

The presentation of lower eyelid concerns from patients who seek treatment for cosmetic improvement is very common. Although the issues are similar (not identical) from patient to patient, the chief complaint from these individuals varies from “sagging of lower eyelids,” lower eyelid bags, “dark circles,” and more recently, lower eyelid “hollows.” For over 100 years, these issues have been treated primarily with surgery.^1^ Unfortunately, many traditional methods for surgical correction (and these are extremely varied, with the approach dependent on a surgeon's biases and preferences) leave the patient with improvement, yet oftentimes with the appearance of having had surgery.^2,3^ This lends itself to patient concerns, which may cause the patient to pause before embarking on a surgical journey for fear of “looking different.” Since the introduction of the first hyaluronic acid (HA) injectable gel filler in the United States, Restylane,^4^ synthetic filling with injectable HA gel agents has been used in an attempt to improve these lower eyelid conditions in many ways to avoid or delay surgery.^5-7^ Before synthetic fillers came into vogue, autologous injectable fat was used but with extremely varied results.^8,9^ The issue related to problems with using autologous injectable fat was the inconsistency and uniformity of results with this approach; oftentimes leading to dramatic surface contour abnormalities.^10^ These issues, when they occurred, were more often treated with a surgical excision of visible fat, which was quite challenging due to the multiple planes of residence of the injected fat offered by the surgeon. The introduction of the use of HA gel fillers was a hopeful attempt to simulate the results that could be obtained with autologous fat for those patients not yet interested in surgery and still provide the possibility of uniformity of results and the potential for complete or partial reversal/removal of the HA product with the use of hyaluronidase if unfavorable outcomes ensued.^11^

Now with more filler options in the US market, many more providers for injectable fillers, and the fact that lower eyelid issues have become a significant presenting complaint of our patients, many more treatments have been performed, unfortunately, with extremely varied outcomes.^12,13^ Adverse events can be related to product selection and technique. Confounding variables also include placement of the product in particular areas/planes of the lower orbit and volumes (amounts) used. We have learned that some products are simply more forgiving, some cause dramatic and predictable lower eyelid edema, whereas others are more sensitive to dissolution with hyaluronidase.^14-16^

To date, the author has treated more than 70 patients with chronic lower eyelid and cheek edema, who had initially presented to their treating physician with a myriad of lower eyelid complaints, were diagnosed with “lower eyelid hollows,” and were treated with HA gel fillers of varied products and techniques. Most of these patients returned to their treating physician requesting treatment (mostly for removal of the product) because of adverse issues, including visibility of the product, chronic persistent or intermittent lower eyelid and malar/cheek swelling, and levels of discomfort. Most of them reported only minimal success despite sometimes more than 6 attempts by the physician to remove the filler using this enzymatic degradation. Patients had presented with concerns ranging from as early as several weeks after the initial filler treatment to more than 10 years posttreatment. Most had no knowledge of which HA product was injected. The author's treatment for a particular subset of problems that relate to chronic lower eyelid and malar edema involves an intraoperative injection of high-dose hyaluronidase with lower blepharoplasty. Outcomes have been extremely favorable. All patients demonstrated significant levels of improvement and most received the outcomes they were originally hoping for.

## METHODS

From June 2016 to present, 70 consecutive patients, 65 females and 5 males, with ages ranging from 31 to 76 years, presented with unsatisfactory results after they received an injection of a HA product elsewhere. Their chief complaint related to “lower eyelid swelling,” and the evaluation confirmed the presence of lower eyelid and/or malar edema combined with typical lower periorbital aging (fat herniation and varied degrees of skin, muscle, and tendon laxity). Most patients in this series presented to the author's practice more than 1 year after treatment with HA elsewhere. Several presented within months of treatment and were advised to return to the treating physician for further treatment (reversal attempt). Seven patients presented more than 4 years after treatment and 2 patients claimed to have been injected with HA to the lower eyelid over 10 years before their presentation. Patients had been treated either once with HA for “tear trough” correction or on multiple occasions. Some presented back to the treating physician with concerns of swelling, and additional HA gel was injected as a proposed remedy. Patients were treated either once or multiple times by the original treating physician in an attempt to “dissolve” the filler after the initial HA treatment, resulting in minimal or no improvement. Some treating providers did not attempt to remove the product, whereas others explained to their patients that the use of hyaluronidase would remove their existing native HA, and therefore, the patients declined treatment. Several presented with a single or a few attempts of lower doses (<20-30 units) of hyaluronidase by the original treating provider and were then additionally treated by the author with extra doses of hyaluronidase (office), resulting in either partial reversal or no detectable reversal of the presenting problem. None of the patients in this series exhibited clinical evidence of granuloma formation or potential biofilm issues. Patients exhibited similar profiles of chronic lower eyelid and malar edema and worsening of their lower eyelid appearance after HA injection administered elsewhere. A detailed discussion with each patient regarding this problem was followed by appropriate informed consent regarding the lower blepharoplasty procedure that would be most appropriate, combined with an intraoperative injection of high-dose hyaluronidase and surgery. Dosing was variable, with an estimate of the amount required to elicit a positive response, and varied from 100 to 200 units per lower eyelid. These estimates considered previous hyaluronidase treatments and their effects, the presence of visible or palpable remaining product, the amount of edema, and on some occasions, the amount of reported volume and which product was used. The hyaluronidase (Hylenex—recombinant hyaluronidase human injection; Halozyme Therapeutics, San Diego, CA) was administered with the anesthetic mixture (2% zylocaine with epinephrine 1:200,00 combined equally with bupivacaine 0.75%) through a transconjunctival approach (to target the postseptal region) at the onset of the procedure that was performed under intravenous sedation if lower blepharoplasty was to be performed or hyaluronidase diluted with nonpreserved saline if lower blepharoplasty was not being done simultaneously (several patients only underwent upper blepharoplasty).

**Figure 1.**
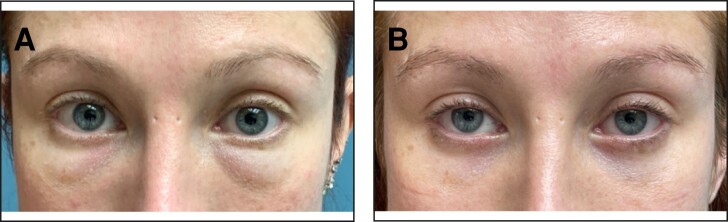
(A) This 35-year-old female patient presented after treatment elsewhere for “lower eyelid hollows” with hyaluronic acid (HA). The patient presented with photographs that confirmed that the situation worsened dramatically after HA treatment. The lower eyelids exhibited not only fat herniation but also a diffuse dark hue without obvious delineation of the lower periorbital fat pads, which was an evidence of chronic lower eyelid edema. (B) The same patient 1 month after a bilateral transconjunctival injection of hyaluronidase (100 units per side) and transconjunctival lower blepharoplasty.

**Figure 2.**
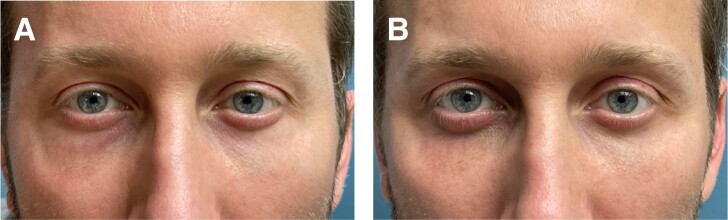
(A) This 38-year-old male patient presented after receiving multiple injections of hyaluronic acid to (mostly) the right lower eyelid elsewhere in an attempt to treat the perceived lower eyelid hollows. He noticed issues after his first injection (mostly edema), and it was suggested that the treatment, to which he had agreed, was to “add more fillers.” (B) The same patient several weeks after 100 units of hyaluronidase were injected to the right lower eyelid combined with bilateral transconjunctival fat reduction.

**Figure 3.**
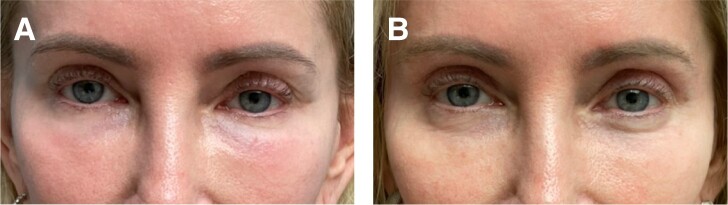
(A) This 50-year-old female patient had an injection of lower eyelid hyaluronic acid several years ago; she presented for undergoing upper and lower blepharoplasty. The upper eyelids exhibited minimal lateral “hooding”/dermatochalasis and lower eyelids showed primarily significant edema. (B) The same patient 2 months after upper blepharoplasty and an intraoperative injection of 100 units of Hylenex to each lower eyelid through a transconjunctival/postseptal approach.

**Figure 4.**
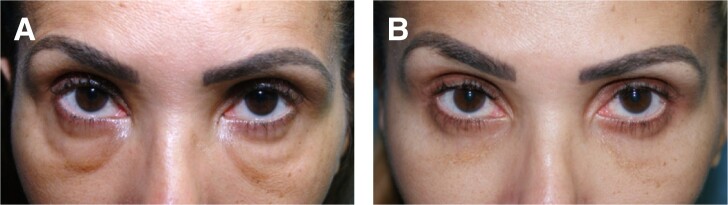
(A) This 50-year-old female patient presented after hyaluronic acid injections were given to her lower eyelid to treat lower eyelid “hollows.” The patient noted a marked lower eyelid and cheek edema and worsening of her condition (“bags”) 1 week after her treatment. Five attempts at dissolution by the treating clinician were in vain. (B) The same patient 3 months after an intraoperative injection of high-dose hyaluronidase (100 units per side) with an extended lower blepharoplasty with lateral retinacular suspension canthoplasty.

Some of the patients had no surgery previously (Figures 1-3), whereas others had a previous lower blepharoplasty with varied procedures performed by surgeons elsewhere (Figure 4). Younger patients with good skin quality and the appearance of lower eyelid fat herniation with the presence of edema (Figures 1, 2), or any patients who had lower blepharoplasty previously with any form of skin tightening, including skin resection (Figure 4), were treated with an intraoperative injection of hyaluronidase (Hylenex), followed by transconjunctival fat contouring (conservative fat excision through a postseptal approach), to resolve both the chronic edema supposed to be related to the persistent residence of HA and the removal of the contour abnormality related to fat herniation. Similarly, patients with acceptable or satisfactory lower blepharoplasty previously were treated with high-dose hyaluronidase and transconjunctival fat contouring. Several patients who presented with upper and lower blepharoplasty exhibited only upper eyelid dermatochalasis and lower eyelid edema and were treated by intraoperative lower eyelid transconjunctival/postseptal high-dose hyaluronidase with upper blepharoplasty (Figure 3). Other patients who had not had blepharoplasty previously and who had significant skin and muscle laxity received interoperative HA injections combined with a lower blepharoplasty (Figure 4) described by the author utilizing a skin flap combined with the orbicularis muscle in lateral retinacular suspension.^17^ There was no attempt, other than typical routine irrigation during a surgical procedure, to physically remove any HA material which, at best, was scant.

This study was conducted in compliance with Good Clinical Practices and is consistent with the ethical principles that have their origin in the Declaration of Helsinki. All patients provided informed consent for surgery.

## RESULTS

No intraoperative or postoperative complications were encountered by the patients. Intraoperative findings were quite variable from the obvious presence of scant clear gelatinous (HA) product mixed with the substance of all subdermal tissue planes, with the absence of any visible material. Many patients who had not had any previous surgery had a clinical observable intraoperative evidence of fibrosis in which the fat pads lacked an unviolated appearance, oftentimes with more difficulty encountered in the dissection or with the typical immediate prolapse during the initial transconjunctival incision that is common in the lower eyelids. The presence of either of these varied findings did not seem to appreciably influence the outcome. There was no clinical evidence of granuloma formation in any of these patients clinically or intraoperatively. All patients had variable but significant reduction of both the chronic edema and the surface contour abnormalities that related to the presumed presence of a persistent product (even when the visible product was not evident or observed during surgery, despite attempts made for enzymatic degradation), chronic edema, and lower eyelid aging (fat herniation).

Follow-up and treatment response assessment were done at 1 month and up to 7 years.

All patients experienced significant improvement in their conditions at varying degrees (from 50% to 100%) and were also mostly satisfied with the treatment approach. Patient descriptions and details of each of these are provided in the legend of each figure. (Figures 1-4)

## DISCUSSION

The treatment of lower eyelid cosmetic displeasures has been a controversial subject long before the advent of injectable HA gel fillers. Debates on what constituted the best surgical treatment for what was then called “lower eyelid bags” ranged from skin flap^17^ or skin–muscle flap^18^ approaches to anterior access to the lower eyelid fat or combined transconjunctival and anterior skin flap approaches (pioneered by the author),^17^ fat “transposition”/“repositioning,”^19,20^ “septal reset,”^21^ autologous fat injection or fat transfer,^8-10,22^ or various forms of (non-HA) injectables,^23,24^ and beyond. The results regarding any of these procedures were highly dependent on the skills of the surgeon, patient presentations, and a matching of the best approach to each individual problem rather than a “one-size-fits-all” approach. Most experienced surgeons will admit that lower eyelid surgery can be quite challenging to the extent that some will even avoid this procedure in their patients.

Because of the variability of surgical results, the high number of unfavorable results, the unwillingness of some surgeons to perform lower blepharoplasty, and the fact of many nonsurgeons/non-MDs entering the cosmetic arena, nonsurgical options (including an injection of HA) have become immensely popular. If all these are combined with the explosive growth of the internet and social media, and the demand from patients for nonsurgical options, it should not surprise us that we have been seeing and will see more patients with unfavorable outcomes. The variability of surgical results can be broken down into several categories: a visible product with obvious contour irregularities; late immunologic reactions (including “biofilm” and granulomata); chronic lower eyelid and malar edema; and patient claims that “eyelid bags seem worse” sometimes many months after treatment. Our report concentrates primarily on the latter. Chronic lower eyelid and malar (cheek) edema and perceived worsening of lower eyelid contours are the most common presentations in our practice among patients who present for cosmetic improvement and have had HA injections previously.

Conceptually, the idea of “filling” makes some sense because the “hollows” that are perceived are essentially shadows created by light that strikes a more cephalad contour convexity (orbital fat herniation), making the hollow look even deeper and dark (ie, dark circles). The problem is that we have yet to arrive on a filler that mitigates all potential issues related to their residence in the thin (thinnest in the body) lower eyelid skin where the distance from the skin surface in the so-called tear trough is but a few millimeters in most patients. Most fillers are variably hydrophilic, and a thin lower eyelid skin, in part, cannot deal with hydrophilicity in the same way as other areas of the face do (nonocular areas may have some advantages). This is particularly true if a patient exhibits any form of preexisting malar edema wherein filling with HA typically will make this situation worse because of the nature of product performance. Furthermore, the concept of the “eraser” that is constantly regurgitated at the podium from proclaimed “experts” stating that unfavorable outcomes (and they position this to their colleagues and to their patients) can be easily reversed reveals the complete ignorance and misunderstanding of what hyaluronidase does. The complexities of hyaluronidases ex vivo and in vivo are difficult to comprehend despite the existence of well-articulated publications, particularly from DeLorenzi.^25^ Hyaluronidase does not “erase” the product; it simply makes smaller oligosaccharide segments that potentially can make it easier for auto-digestion which in itself could pose a different problem beyond the scope of this discussion. My presumption and experience is that even smaller HA fragments still elicit a hydrophilic response and further, repeated injection of hyaluronidase (as is common) may yield only limited improvement. One commonly noted issue after an untoward effect after HA injection is the patient complaint (oftentimes demonstrated with selfie photographs) that the lower eyelid bags seem worse after treatment. Sometimes this relates to preexisting malar edema that becomes worse; but we have consistently seen photographic evidence (patients will present with confirmatory photographs taken before the time of the HA lower eyelid treatments) of lower eyelid bags and skin laxity degradation after multiple treatments of HA to the lower eyelid. Possibilities for causation here include a low-grade inflammatory response to either the product or the “break-down” products (i.e., with unsuccessful repeated attempts to “dissolve” the filler with hyaluronidase) with further herniation or visibility of the lower eyelid fat pads, but this is still unclear. This is also another reason why in some patients with this phenomenon, hyaluronidase alone will not result in a favorable outcome. Another issue is that many injectors more often administer subtherapeutic amounts of hyaluronidase because of either a misunderstanding of the amount of dosing required for an improvement (C. DeLorenzi, personal communication, July, 2022) or an erroneous and misinterpreted concern that hyaluronidase will cause irreversible diminishment of a patient's native HA.^25,26^ It has been the experience of the author that, in many situations, at least 100 units of hyaluronidase for each lower eyelid is required to significantly and positively influence the outcome. Finally, a strong and prevailing concept that has validity and also conceived by DeLorenzi (personal communication, 2022) is that some injected HA that is nonresponsive to hyaluronidase emanates from a subseptal (posterior to the orbital septum) residence of the product, as evidenced by the initial failure with superficial hyaluronidase injection and the subsequent success with a deeper, postseptal application. The orbital septum may be impervious to hyaluronidase, and attempts at “rescue therapy” are usually made preseptally, which will have only minimal effect. This, of course, makes complete sense, and we agree on this, but the author can attest to the fact that in some patients who present after multiple attempts to inject hyaluronidase deep into the septum, the chronic edema persists. This suggests that an alternate approach may be required. The author follows this presumption by administering the hyaluronidase with the local anesthetic in a transconjunctival/postseptal injection at the onset of the surgical procedure.

In addition, the author has used this approach of combination hyaluronidase/surgery on several patients who have previously had traditional lower blepharoplasty (Figures 1, 2) with a skin–muscle flap approach with either removal or disruption of the lower orbital septum by the original surgeon and were then treated years later with HA fillers, resulting in lower eyelid edema, wherein multiple in-office attempts to dissolve the HA was unsuccessful. Previous reports on the successful surgical treatment of unsatisfactory lower eyelid filler-related issues have focused more on the direct filling effect (contour irregularities created by the residence of the HA) and surgical correction but not on other related adverse events such as chronic, persistent edema.^27^ Others also have discussed the management of removing the HA filler before surgery for better outcomes.^28^

Of course, this has yet be proven, but the consistent reversal utilizing a combination of high-dose hyaluronidase and surgery lends itself to a reasonable conclusion that some HA remains (oftentimes for many years and despite multiple attempts with office-based treatments with hyaluronidase), and the inflammatory response to surgical healing may be the “secret sauce” to a complete resolution of the presenting problem. The author believes that there can be many factors that influence the positive results achieved here. One can speculate that some HA essentially remains “immune” or nonresponsive to additional hyaluronidase and there is no (or a suboptimal) mechanism for the removal of the remaining substance that is better addressed with the addition of surgical trauma (blepharoplasty) and the inflammation with all of the inflammatory mediators and cellular response (macrophages, etc) to remove this foreign material. In some patients, breaking down the HA molecule may not be enough to elicit this necessary response. Furthermore, the mechanical effects of persistent HA on the local lymphatics to effectively “drain” these areas has been a subject of debate; it is still unproven,^29^ but may likely also be a factor that is reversed with effective treatment. The time from initial treatment to the attempt to reverse adverse events will likely influence the outcome. Chronic malar edema from injected HA that has persisted for many years may be less likely to be completely reversed because of the creation of large potential spaces that do not significantly diminish with treatment. Finally, the administration of transconjunctival (postseptal) hyaluronidase at the onset of the procedure, done in an office setting by an experienced surgeon, might significantly influence the result in some patients, but it has been the author’s experience that the combination of transconjunctival high-dose hyaluronidase with lower blepharoplasty seems to more thoroughly reverse adverse events in a majority of patients.

## CONCLUSIONS

The combination of an intraoperative injection of high-dose hyaluronidase and (upper and/or) lower blepharoplasty can lead to satisfactory outcomes in patients with chronic lower eyelid edema after the administration of HA gel injections to the lower eyelid. As expected, not all patients will respond as effectively as some because of a myriad of predisposing factors. Adverse events including chronic persistent lower eyelid and malar/cheek edema will become more common as a presentation of patients seeking cosmetic improvement of the lower periorbita who have received HA injections to this region. It is expected that newer products that consider these inevitable effects will arrive that will hopefully address and obviate these concerns. Nonetheless, existing products will continue to be used, and the practitioner should be aware of potential treatments that can lead to successful outcomes.
